# Stradivari’s Varnish Revisited: Feature Improvements Using Chemical Modification

**DOI:** 10.3390/polym15173652

**Published:** 2023-09-04

**Authors:** Maduka L. Weththimuni, Giacomo Fiocco, Chiara Milanese, Alberto Spinella, Maria Luisa Saladino, Marco Malagodi, Maurizio Licchelli

**Affiliations:** 1 Department of Chemistry, University of Pavia, Via T. Taramelli 12, 27100 Pavia, Italy; chiara.milanese@unipv.it; 2 Research Center for the Conservation of Cultural Heritage (CISRiC), University of Pavia, Via A. Ferrata 3, 27100 Pavia, Italy; giacomo.fiocco@unipv.it (G.F.); marco.malagodi@unipv.it (M.M.); 3 Department of Musicology and Cultural Heritage, University of Pavia, Corso Garibaldi 178, 26100 Cremona, Italy; 4 Advanced Technologies Network Center, University of Palermo, Viale delle Scienze, Ed. 18, 90128 Palermo, Italy; alberto.spinella@unipa.it; 5 Department of Biological, Chemical, and Pharmaceutical Sciences and Technologies (STEBICEF), University of Palermo, Viale delle Scienze, Ed. 17, 90128 Palermo, Italy; marialuisa.saladino@unipa.it

**Keywords:** Stradivari, violin varnishes, linseed oil/colophony, SEM-EDS, NMR

## Abstract

The most widespread varnish formulations used by master violin-makers of the “Italian Golden Age”, including Antonio Stradivari, were based on mixtures of siccative oils (e.g., linseed oil) and natural resins (e.g., colophony). Similar formulations are still used for the finish of contemporary instruments. Although most precious violins made by Stradivari and other Cremonese Masters are kept in museums, several instruments are still played and their finish may undergo deterioration due to contact with the players. Moreover, the decay of the traditional varnish may occur due to mechanical stress and natural aging caused by environmental agents (e.g., exposure to uncontrolled light, humidity, and temperature changes). The main aim of this research work is to investigate the possible improvement of varnish resistance to the decay induced by different aging processes. For this purpose, the traditional varnish (linseed oil/colophony 3:1 *w*/*w*) was recreated in the laboratory following an ancient recipe and then it was functionalized with a cross-linking agent (3-Glycidyloxypropyltrimethoxysilane, GLYMO). Plain and functionalized varnishes underwent artificial aging (UV light, temperature, and humidity variations), and their properties were comparatively studied using different techniques. All the results suggest that the functionalized varnish displays improved resistance to the aging process and particularly enhanced photostability and increased hardness (resistance to scratches).

## 1. Introduction

String musical instruments are traditionally coated with natural varnishes (of animal or vegetable origin), which protect them from light, dust, humidity, and climatic changes and improve the mechanical properties and aesthetical appearance of their surface [1]. Moreover, the existence of a direct relationship between the composition of the varnish and the quality of the sound produced by the instruments is almost certain [2,3].

Ancient instrument-makers used three main varnish types, commonly named spirit varnishes, essential oil varnishes (volatile oil), and oil varnishes (fixed oil-non-volatile) [1,4]. Since the 16th century, the principal binders for the preparation of varnishes have been siccative vegetable oils because of their unique physico-chemical properties and good performances in the surface finish (e.g., high gloss and transparency) [5]. Siccative oils are mainly made by triglycerides of unsaturated fatty acids, i.e., oleic, linoleic, and linolenic, which contain one, two, and three double bonds, respectively [6]. Upon exposition to air, they undergo a chemical reaction, known as auto-oxidation [7], involving the spontaneous addition of oxygen to the unsaturated acyl chains to form unstable hydroperoxide compounds. Radical species originating from hydroperoxide decomposition promote a variety of further chemical processes, including intermolecular couplings, which induce the formation of a polymeric network responsible for the drying of the oil films [6,8].

Several research studies suggest that the most widespread varnish formulations used by master violin-makers, including Stradivari, for the finishing of bowed musical instruments were based on mixtures of linseed oil (the most popular siccative oil) and natural resins, especially Pinaceae resins (e.g., colophony), in the period between the 17th and 18th century (the so-called Golden age of the Italian luthiers) [1,9,10]. 

Colophony is a natural product mainly consisting of various resin acids, especially abietic acid [11,12], which has been widely used as a component of varnishes due to its excellent properties (fast drying, excellent solubility and compatibility with other resins and oils, and ready availability). Fascinated by the uniqueness of instruments made by the great Masters of the past, particularly Stradivari’s violins, contemporary luthiers have rediscovered the goodness of historical varnishes, also experimenting with oil/resin mixtures of different compositions. These mixtures have been studied in recent years, and the results have shown that the composition of 3:1 (oil:resin) would still provide a varnish displaying the highest quality and durability, especially in the case of the ancient musical instruments still played today [13].

Some researchers have focused their interest on this field to address some conservation issues concerning historical musical instruments. Several of them, particularly the precious violins made in the 17th and 18th centuries (e.g., by Stradivari and other Cremonese Masters), even if kept in controlled museum environments, are still played, and may undergo deterioration due to different factors: (i) contact with the violinist’s skin and perspiration (pH < 7), (ii) exposure to sunlight and in particular to UV components, humidity, and temperature changes. As a consequence, these phenomena may cause drastic damage (exfoliation, particle removal, fading, or browning) to the varnish layer [4]. 

Therefore, appropriate solutions or methods to overcome varnish declines and to better protect these precious musical instruments are highly desirable. The historical instruments under consideration represent an invaluable heritage from a cultural, artistic, and economical point of view. In addition, research studies addressing how to avoid or reduce the weaknesses of Stradivari’s varnish could be of great help to contemporary violin-makers who use the traditional finishing method.

In fact, the main aim of this research work is to perform chemical modifications of the so-called Stradivari’s varnish to improve some of its properties (e.g., hardness, photostability) without affecting its good finish performance. In particular, the effect of additional cross-linking induced using an epoxysilane derivative (3-Glycidyloxypropyltrimethoxysilane, GPTMS also known as GLYMO) has been investigated. 

GLYMO is a bifunctional organosilane containing a reactive organic epoxide and hydrolyzable methoxysilane groups. The dual nature of its reactivity allows it to chemically bind to both inorganic and organic compounds, thus acting as a cross-linking agent and/or surface modifier. 

In the present work, Stradivari’s varnish was prepared in the laboratory following an ancient recipe [14,15], which also involves the heating of linseed oil and colophony at quite a high temperature (270 °C). The mixture was then treated with GLYMO to obtain chemical functionalization. Furthermore, both plain linseed oil and colophony were also reacted separately with the same epoxysilane to better understand the possible reaction(s) taking place in the mixture. After application to wood specimens in the laboratory, the properties and performances of the modified varnish were studied in comparison with the traditional one using a wide range of techniques, such as chromatic variations and contact angle measurements, micro Fourier-transform infrared spectroscopy (μ-FTIR), optical microscopy (OM), scanning electron microscopy (SEM), energy-dispersive X-ray spectroscopy (EDS), thermogravimetric analysis and differential scanning calorimetry (TGA and DSC), nuclear magnetic resonance spectroscopy (NMR), and hardness measurements. The behavior of varnishes (traditional and modified) was investigated after exposing coating films and coated wood specimens to artificial aging processes induced by UV irradiation and thermal treatment. 

A proper comparison of results obtained on aged and unaged materials suggests that the addition of limited amounts (about 5%) of GLYMO to the traditional varnish induces considerable improvements in its performance, including enhanced resistance to mechanical decay and to aging. 

## 2. Experimental Section

### 2.1. Materials

Linseed oil (n73100 Kremer, Kremer Pigmente GmbH & Co. KG, Aichstetten, Germany) containing Mn-based catalyst (to accelerate the drying process) and colophony were purchased from Kremer-Pigmente (Aichstetten, Germany). Both products were used without any further purification for the preparation of the investigated varnish (75/25 *w*/*w*, linseed oil/colophony). Moreover, 3-glycidyloxypropyltrimethoxysilane (GLYMO, ≥98%), dibutyltindilaurate (C_32_H_64_O_4_Sn, DBTDL, 95%), anhydrous ethanol (C_2_H_5_OH, ≥99.8%,) and deuterated chloroform (CDCl_3_, 99.8 atom% D) were supplied by Sigma-Aldrich (St. Louis, MO, USA); dichloromethane (CH_2_Cl_2_, 99.5%, stabilized with about 0.2% C_2_H_5_OH) was provided by Carlo Erba (Milan, Italy); and n-octane (C_8_H_18_, 95%) was purchased from Sigma-Aldrich (St. Louis, MO, USA) and used for cleaning the brushes between paint applications.

The maple wood specimens (5 × 5 × 0.5 cm^3^) used in the present work were kindly provided by Civica Scuola di Liuteria (Milan, Italy), while the brushes (Habico-204 AK, Cremona tools, Cremona, Italy) with wild boar bristles for the application of paints on wood and glass slides were purchased at Cremona-Tools. Abrasive papers (P400 and P800 Abranet ^®^, Mirka Italia srl, Tuscany, Italy) were used for cleaning the wood specimens. 

### 2.2. Methods

#### 2.2.1. Preparation and Application of Varnishes and Other Materials

Stradivari’s varnish (72/25: linseed oil, L/colophony, C) was prepared in the laboratory following an ancient recipe [14,15] as previously described [4] and named varnish LC. 

The silane-modified material named varnish LCS was prepared by reacting varnish LC with GLYMO according to the following procedure, which was selected after conducting several trials and according to our previous experience with different varnish modifications [16]: 3-glycidyloxypropyltrimethoxysilane (1.5 mL) was added into a round-bottom flask containing varnish LC (30.0 mL) and then the reaction mixture was heated until 60 °C in the presence of dibutyltindilaurate (DBTDL, 0.09 mL), which acts as a catalyst. The reaction was carried out under an N_2_ atmosphere for 24 h. At the end of the reaction, varnish LCS was collected and stored in a glass bottle.

In addition to the preparation of the two main varnishes (LC and LCS), pure components of LC were also reacted with epoxysilane to study its possible reactions with oil components as well as with colophony. The resulting compounds were named LS and CS, respectively.

Material LS was obtained by reacting plain oil L with epoxysilane according to a similar procedure to that conducted for LCS. 

For the preparation of functionalized colophony (CS), colophony (5.0 g) was dissolved in anhydrous ethanol (1:2 volume), then GLYMO (0.25 mL) was added, and the reaction was performed according to the experimental conditions described for LCS. The solid product was recovered after solvent evaporation at room temperature under a fume hood for several days.

Varnish films (LC and LCS) were prepared on glass slides (microscope slides) using flat brushes made from boar hair because these kinds of varnishes are self-leveling. In order to obtain the correct thickness of the film, five consecutive applications (five layers) were performed after drying each layer. The full drying of films of varnishes was obtained after 7 days at room temperature (20 ± 2 °C) and 2 days under a UV lamp (UV box: two lamps of Philips TL-D 36W BLB with an emission peak at 360 nm). Thirty-six varnish films were prepared in both kinds of varnishes, and for each experimental analysis, three films of the same treatment were used to obtain the correct results. 

Furthermore, both varnishes (LC and LCS) were applied to previously cleaned (using two kinds of abrasive papers: P 400 and P 800) maple wood specimens (48 wood samples) using the same brushes (flat brushes made from boar hair) according to the real cases, as explained clearly in our previous papers [17,18]. To obtain the correct thickness of the coating, eight applications were carried out in perpendicular directions for the wood specimens, and the drying process was conducted in the same way as in the varnish film preparation. Moreover, three different wood specimens of each treatment (LC and LCS) were used for each experimental analysis in order to calculate the average value of the measurements.

#### 2.2.2. Instruments and Techniques

Infrared spectra were collected using both a PerkinElmer Spectrum 100 FT-IR instrument (Perkin-Elmer, Waltham, MA, USA), equipped with a universal ATR accessory (Diamond crystal), and a Nicolet iN10 Thermo Fischer μ-FT-IR spectrometer (Thermo-Fisher, Waltham, MA, USA) in ATR mode (Germanium crystal). The thermogravimetric measurements (TGA) were performed with a Q5000 (TA Instruments, New Castle, DE, USA) system under an air flow (10 mL/min) in a platinum sample holder. Here, a few milligrams of samples were heated from 25 °C to 1000 °C at a rate of 10 °C/min. Moreover, the scanning differential calorimetry analysis (DSC) was performed with a Q2000 instrument (TA Instruments) by heating a few milligrams of sample in an open aluminum crucible from −90 °C to 300 °C, at a speed of 10 °C/min, under air flow (50 mL/min). Chromatic variations were measured using a Konica Minolta (Konica Minolta, Inc., Tokyo, Japan) CM-2600d spectrophotometer, determining the L*, a*, and b* coordinates of the CIELAB space and the global chromatic variations ΔE* according to the UNI EN 15886 protocol [19]. Five measurements on each wood specimen were carried out, and the collected results are average values from fifteen measurements (three different wood specimens were used for each treatment) taken before treatment, after treatment, and after aging. 

Water contact angle measurements were carried out using a Lorentzen and Wettre instrument (Zurich, Sweden) according to the UNI EN 15802 Protocol [20]. The results were collected as average values by taking 15 different measurements for each kind of treatment (three different wood specimens were used for each coating). The optical microscope observations were conducted using a light polarized microscope, equipped with an Olympus TH4-200 lamp (visible light). Scanning electron microscopy (SEM) images (backscattered electron) and energy dispersive X-ray spectra (EDS) were collected by using a Tescan FE-SEM, MIRA 3XMU series (Tescan, Brno, Czech Republic) equipped with a Schottky field emission source, operating in both low and high vacuums and located at the Arvedi Laboratory, CISRiC-University of Pavia, Italy. Before SEM analysis, samples were gold-sputtered using a Cressington sputter coater 208HR (Ted Pella, Inc., Redding, CA, USA). 

Hardness measurements were determined by pencil test according to the ISO standard (ISO 15184:1998) [21], and the obtained results were average values from three different measurements (three different wood samples of each treatment were used for the test).

#### 2.2.3. NMR Experiments

For liquid-state NMR, all samples were dissolved in deuterated chloroform (CDCl_3_). The spectra were recorded at 300 K by means of a Bruker Avance II 400 spectrometer operating at 400.15 and 100.63 MHz for the ^1^H and ^13^C nuclides, respectively, equipped with an inverse broadband (BBI) probe. 

^1^H spectra were acquired using a 12.15 µs pulse, a delay time of 3 s, and 16 scans.

Solid-state NMR experiments were performed using the spectrometer described above using a 4 mm H-X CPMAS probe. ^13^C CPMAS spectra were acquired with an MAS spinning speed of 8 kHz, 1024 scans, a contact time of 1.5 ms, a delay time of 3 s, and a ^1^H pulse of 4.5 µs. The optimization of the Hartmann–Hahn condition was obtained using an adamantane standard sample. This compound was also used as an external chemical shift reference. All samples were placed in zirconia rotors sealed with KEL-F caps.

The ^1^H spin-lattice relaxation time in the rotating frame (T_1ρ_H) was obtained using the variable spin lock (VSL) sequence using spin-lock pulses ranging from 0.1 to 7.5 ms with a spin lock B1 field of 57.6 kHz and a contact time of 1.5 ms.

The ^13^C spin-lattice relaxation time in the rotating frame (T_1ρ_C) was obtained using the variable spin lock (VSL) sequence using spin-lock pulses ranging from 0.4 to 30 ms and a contact time of 1.5 ms.

#### 2.2.4. Artificial Aging of the Samples

When completely dried, the treated wood samples (36 specimens; 18 from each treatment) and glass slides (24 varnish films; 12 from each treatment) were subjected to two different types of artificial aging processes (UV irradiation and thermal/humidity treatment). UV aging was conducted (for 720 h) using an irradiation system (Helios Italquartz, Milan, Italy) equipped with two mercury lamps (power: 15 W), as previously reported [18,22,23], and thermal/humidity aging was conducted in a climatic chamber at a temperature (T) of 45 °C with 28% relative humidity (RH) for 720 h (1 month) [2,24].

## 3. Results and Discussion

### 3.1. Preparation of Plain and Modified Stradivari’s Varnishes

**Scheme 1:**
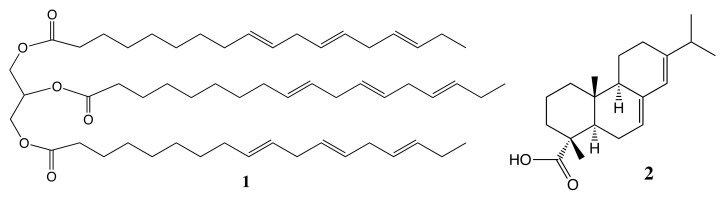
Formulae of tri-linolenic glyceride (**1**) and abietic acid (**2**).

Different investigations carried out over the past few decades suggested that linseed oil and colophony (or equivalent compounds) were the main components of varnishes applied by the greatest violin-makers of the past, and particularly by Stradivari, to their precious instruments [1,12]. Among the different composition ratios proposed based on the experimental results for investigated varnishes, the 75/25 *w*/*w* (oil/resin) ratio has been hypothesized as the one providing the most homogeneous coating. Such a mixture composition roughly corresponds to a 1:1 stoichiometric ratio, if tri-linolenic glyceride (**1**) and abietic acid (**2**, Scheme 1) are taken as representative compounds for linseed oil and colophony, respectively [25].

According to ancient recipes [14,15], blending linseed oil with colophony requires prolonged heating (up to 3 h) at quite a high temperature (up to 270 °C), which induces chemical modifications of diterpenic acids in colophony and (poly)unsaturated triglycerides in linseed oil [26,27,28], including isomerization and oxidation as well as the partial hydrolysis of triglycerides [29]. In particular, oxidation processes may generate ketones, aldehydes, alcohols, and carboxylic acid functionalities [6,8,30,31,32]. Moreover, esterification involving carboxylic derivatives of resin and diglycerides formed by the partial hydrolysis of oil components has been reported [33,34,35,36].

Following this “cooking step,” varnish can be used for application on the wood surface. Its exposition to air and light induces the well-known curing process due to a quite complex radical mechanism that involves unsaturated chains of fatty acids and dioxygen and provides the final coating [6,8,33,35].

The introduction of the silane coupling agent into the siccative oil/resin mixture mainly aims to induce an additional cross-linking in the varnish matrix besides the above-mentioned curing process. It is expected that such an additional chemical process provides an improvement of specific varnish features, such as hardness and resistance to aging. 

**Scheme 2:**
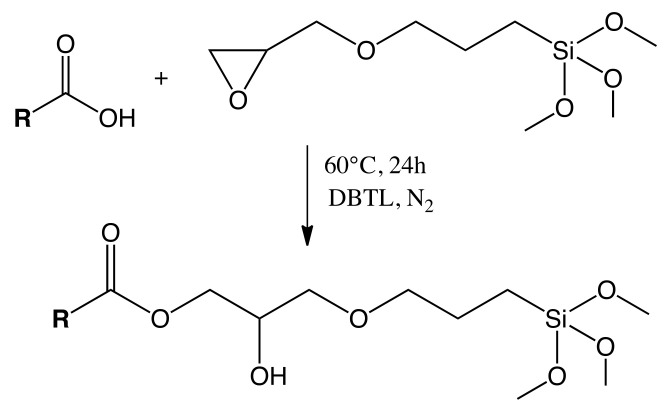
Possible reaction involving carboxylic groups of varnish components with epoxysilane (RCOOH = abietic acid or similar component of resin, unsaturated fatty acid, or oxidation product of triglycerides).

**Scheme 3:**
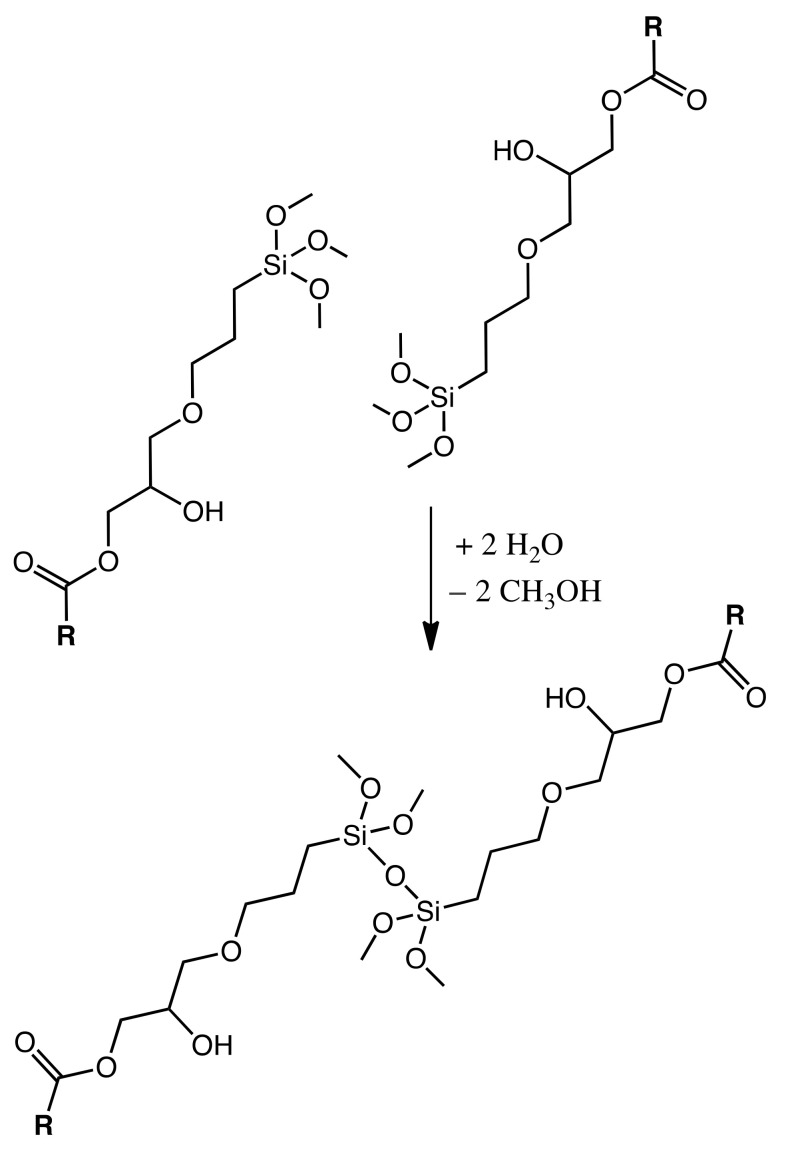
Example of possible condensation reaction involving trimethoxysilane groups introduced on different components of the varnish (RCOO groups deriving from abietic acid or similar component of resin, unsaturated fatty acid, or oxidation product of triglycerides).

Hence, epoxysilane can promote two different reactions involving the varnish components: (i) epoxy groups of silane undergo oxyrane ring opening by carboxylic groups, with the consequent formation of an ester bond (Scheme 2). In the oil/resin mixture, free COOH functions can be provided by colophony components (e.g., abietic acid, **1**) by free fatty acids, and by the other possible oxidized compounds formed during the heating of the oil/colophony mixture; (ii) hydrolysis/condensation of the trimethoxysilane groups (Scheme 3). Reaction (i) is performed in quite mild experimental conditions (60 °C, 24 h) in the presence of a catalyst (DBTDL) and allows us to introduce trimetoxysilane groups into different chemical components of the mixture. It should be noted that this reaction is carried out in the N_2_ atmosphere in order to prevent the oxygen-induced curing of the varnish at this stage. 

In principle, the hydrolysis of trimethoxysilane groups and their further condensation can occur, at least partly, during the functionalization step, although the availability of water in such an experimental environment should be very limited. Therefore, it is expected that silane-induced cross-linking mainly occurs after the exposition of varnish to air (and ambient humidity), when the conventional curing process takes place. Moreover, epoxy groups that possibly have not reacted in the functionalization step are still allowed to interact with carboxylic groups that form due to oxidation processes during the conventional curing step. 

The products formed owing to alcoxysilane condensation that cause additional cross-linking can be countless. The process illustrated in Scheme 2 represents just an example. In addition, a further reaction between methoxysilane side-chains and -OH groups belonging to cellulose or lignin cannot be ruled out after the application of varnish on the wood surface [36].

### 3.2. Characterization of Varnish Materials before and after Aging

#### 3.2.1. Optical Microscopy Analyses

**Figure 1:**
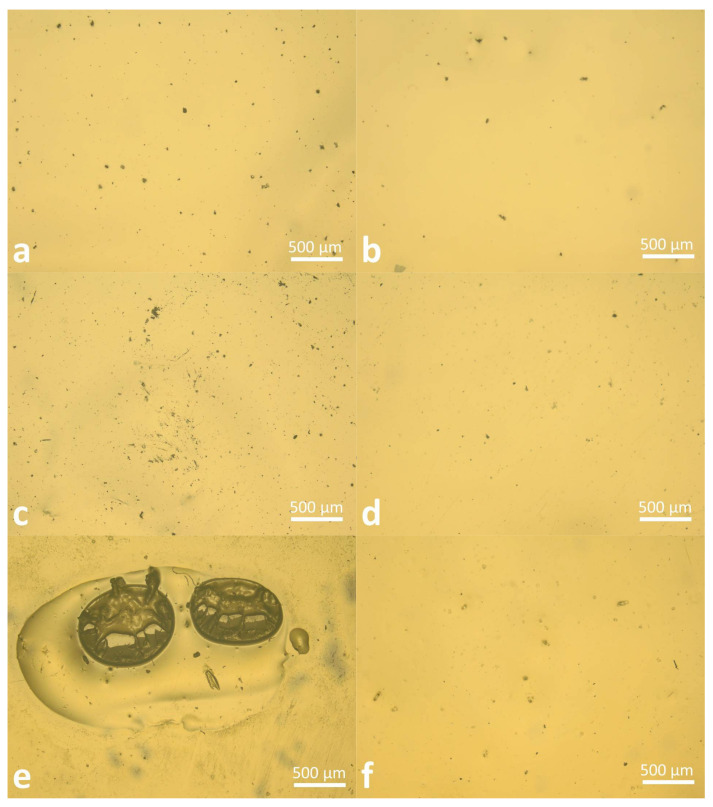
Optical microscope images of varnish LC and varnish LCS films (left and right side, respectively): (**a**,**b**) before aging, (**c**,**d**) after UV aging and (**e**,**f**) after thermal aging.

Films of the original Stradivari varnish (LC) and of the modified one (LCS) were prepared on glass slides according to the traditional curing procedure (see Section 2.2.1) and observed using an optical microscope before and after the artificial aging process to point out the possible morphological differences between the two materials and any variations induced by aging processes. Before aging, the films of the two different varnishes are comparable to each other both when observed by the naked eye and by microscope. In particular, it was not possible to notice any differences from the homogeneity point of view (Figure 1a,b). However, the films of varnish LC show extensive scratches on the entire surface after exposition to UV irradiation (Figure 1c), and a more drastic deterioration was observed after the thermal aging (Figure 1e). In this last case, the varnish film displays a particularly inhomogeneous aspect with the presence of several aggregates. On the contrary, the films of functionalized varnish LCS do not undergo significant alterations as its the surface still appears smooth and homogeneous even after aging (Figure 1d,f).

#### 3.2.2. FTIR Investigations

The FTIR analyses were carried out on coating films to highlight the chemical modifications induced by functionalization on the original components (L and C) and on varnish LC.

**Figure 2:**
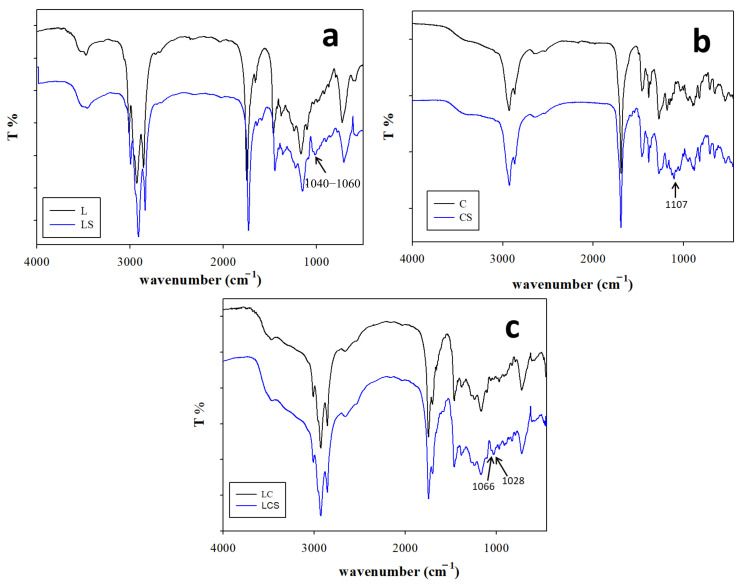
FTIR spectra of original and functionalized materials: (**a**) L and LS, (**b**) C and CS, and (**c**) varnish LC and varnish LCS.

Figure 2 compares the spectra of linseed oil and linseed oil after the reaction with epoxysilane (L and LS, Figure 2a); of pure rosin and functionalized rosin (C and CS, Figure 2b); and of the two varnishes (LC and LCS, Figure 2c). Looking closely at the fingerprint area, some differences can be observed, particularly in the region between 1100 and 1000 cm^−1^. The new peaks in the spectra of silane-functionalized samples, which do not appear in the corresponding plain materials, could be ascribed to the trimethoxysilyl groups (Si-O-C bonds) or to possible siloxane functions (Si-O-Si bonds) [37,38,39,40]. The spectrum of plain epoxysilane (GLYMO) is reported in Figure S1 for comparison.

The presence of Si-O-C and Si-O-Si bonds would confirm the reaction between carboxylic groups and epoxide as well as the possible subsequent condensation of the alkoxysilane groups in the presence of the DBTDL catalyst.

#### 3.2.3. TGA and DSC Measurements

**Figure 3:**
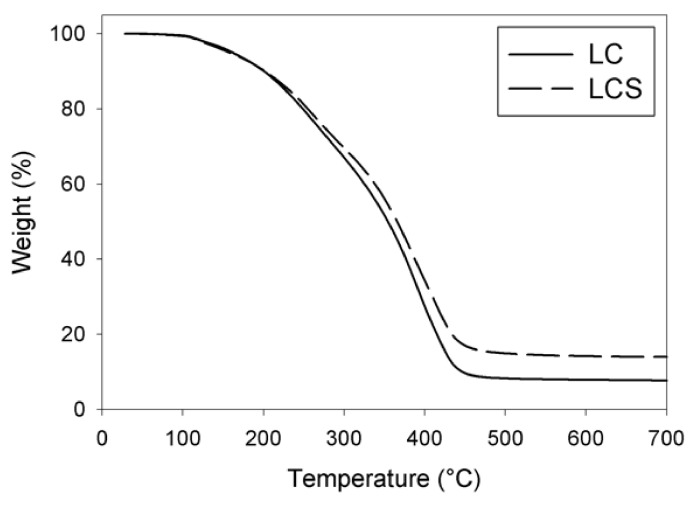
Thermogram (TGA) of varnishes LC and LCS.

Film samples of the considered varnishes (LC and LCS), prepared using the traditional curing process, were also investigated using TGA and DSC to assess the possible variations caused by functionalization on the thermal behavior of the Stradivari varnish and of its modified analog. Thermogravimetric analysis (temperature increasing from 25 °C to 1000 °C) showed that varnish LC undergoes degradation in the 102–460 °C range, suffering an overall weight loss of 93.5%, while the functionalized LCS material decomposes between 106 °C and 500 °C, with a mass loss of 86.9% (Figure 3). These data suggest that the introduction of GLYMO into the varnish matrix caused a small improvement in the resistance to heat degradation (around a 7% difference in weight loss), which could be related to the additional curing process promoted by the epoxysilane additive. TGA experiments were also performed on film samples obtained by plain linseed oil (L) and colophony (C), as well as on their derivatives obtained after the reaction with GLYMO (LS and CS, respectively), in order to verify if the single components of the Stradivari varnish are affected by the functionalization with epoxysilane. 

The decomposition process observed for film L started at 101 °C and end at 475 °C, in accordance with previously published data [4], while the degradation of the functionalized sample LS is observed up to 500 °C (Figure S2). Moreover, the percentages of weight loss after decomposition correspond to 94.8% and 86.4% for L and LS, respectively. The plain colophony sample (C) decomposed between 122 °C and 300 °C, with a final weight loss of 100.0%, in agreement with what was previously reported [4], while the heating of CS induced two decomposition processes in succession: the first was more substantial, in the 80–316 °C range, and generated a weight loss of 83.4%, while the second was between 316 °C and 450 °C and showed an overall weight loss of 98.8% (Figure S3). The two subsequent decompositions can be explained by considering that colophony is only partially functionalized with GLYMO: the most abundant unreacted fraction behaves similarly to plain C, while the less abundant silane-functionalized fraction is more resistant to thermal decomposition.

These data confirm that siccative oil and rosin react with GLYMO during the functionalization process, both as isolate components and when they are blended to form the so-called Stradivari varnish. In any case, the introduction of silane functionalities into their structures significantly affects the thermal behavior of the resulting materials. In particular, varnish LCS displays an improvement in resistance to heat, which can be ascribed to the positive combination of the silane-induced cross-linking and the conventional siccative oil curing process. 

Differential scanning calorimetry (DSC) analyses were also carried out on samples of varnish films (LC and LCS) as well as on plain and functionalized components. In fact, it is expected that structural modifications affect the phase transitions of the investigated materials and particularly the glass transition temperature (T_g_), i.e., the temperature at which the varnish (or the resin) changes from a rigid glassy material to a soft one. The T_g_ values are summarized in Table 1. It should be noted that all materials obtained after the GLYMO-induced modification are characterized by T_g_ values significantly higher than the corresponding unmodified compounds. Specifically, varnish LCS undergoes glass transition at about 40 °C, while the traditional varnish LC has a T_g_ value of about 17 °C, indicating that the varnish modification provided a relevant increment to the glass transition temperature (the value more than doubled) of the not-modified varnish. This means that the traditional varnish material becomes a little bit hard (reduced softness) due to the chemical modification. Similarly, the material obtained by curing plain linseed oil experiences a distinct increase in T_g_ due to the functionalization with epoxysilane (T_g_ = −6.8 and 31.5 °C for L and LS, respectively). Pure rosin, C, showed a T_g_ of 60 °C, in agreement with the literature [41], while the corresponding silane-modified material, CS, undergoes glass transition at 75 °C. 

The study using thermal analyses was also performed on varnishes LC and LCS after their artificial aging. The most interesting results come from DSC experiments and, in particular, from the glass transition temperatures. In fact, the LC material undergoes a drastic T_g_ decrease (by about 40 °C, see Table 1) after the aging induced both by UV irradiation and temperature/humidity variation cycles. This result suggests that the conventional varnish is strongly affected by the aging process, which makes the material softer. The modified varnish, LCS, also experiences a decrease in T_g_ after aging, although the observed variations are distinctly more restrained than in the case of LC. In fact, for the modified varnish, T_g_ decreases from 40.3 to 33.5 °C after UV-induced aging and to 25.5 °C after thermal aging.

Hence, TGA and DSC measurements clearly indicate that the structural modification induced by GLYMO in the varnish matrix provides a more resistant and less soft material, which is also able to better preserve its properties after artificial aging. 

#### 3.2.4. NMR Analyses

The varnishes and their components were also investigated using nuclear magnetic resonance (NMR) spectroscopy both in CDCl_3_ solution (L, LS, and varnishes LC and LCS before curing) and in the solid phase (L, LS, C, CS, and varnishes LC and LCS after curing) in order to gain a better insight into the structural modifications induced by the cross-linking process following the epoxysilane introduction. 

**Figure 4:**
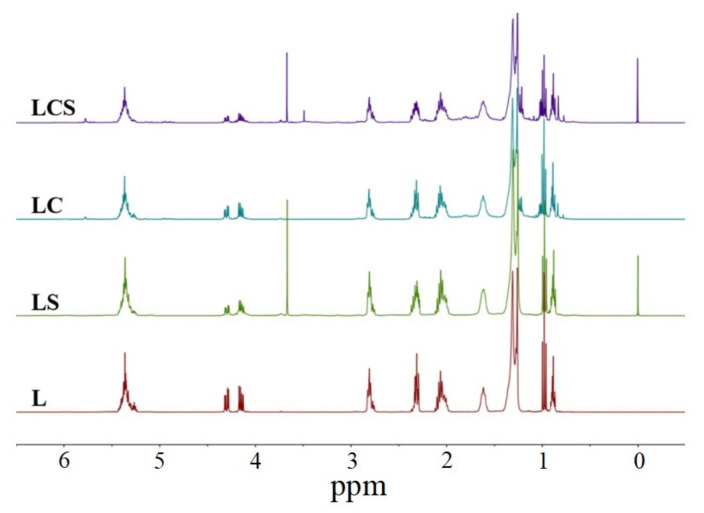
^1^H NMR spectra in CDCl_3_ of plain and functionalized materials.

The liquid-state ^1^H NMR spectra of the original and functionalized materials are reported in Figure 4. Enlargements of the most relevant spectral regions are reported in Figure S4. 

The signals due to linseed oil are present in all spectra. In particular, the resonances between 5.2 and 5.5 ppm are due to the CH of glycerol moiety in triglycerides and to vinyl protons of unsaturated fatty acid chains. Methylene protons of glycerol moiety in triglycerides (and possibly in 1,2- and 1,3-diglycerides) resonate between 4.1 and 4.4 ppm, while the CH_2_ between the double bonds in unsaturated chains resonates at 2.8 ppm. The signals at 2.3, 2.0, and 1.6 can be assigned to CH_2_ in the α position to carbonyl groups, to CH_2_ bonded to one double bond, and to CH_2_ in the β position to the carbonyl groups, respectively. The intense peaks centered at 1.3 are due to all the other methylene groups in aliphatic chains, while the terminal methyl groups of the fatty acid residues resonate between 0.9 and 0.8 ppm.

The spectra of LC and LCS additionally display other signals due to the colophony component. Selected spectral regions of the two varnishes are reported in Figure S5. The spectrum of plain colophony is also reported for comparison. In particular, sharp peaks between 0.75 and 1.25 ppm can be ascribed to methyl groups, while low intensity signals between 4.7 and 6.0 ppm and between 6.8 and 7.3 ppm can be due to the protons of endo- and exo-cyclic double bonds of abietic acid and other similar structures. 

Spectra of the silane-functionalized materials (LS and LCS) display a singlet at 3.7 ppm due to unreacted methoxy groups of epoxysilane. On the other hand, signals ascribable to an epoxy ring, such as the multiplet at about 3.4 ppm (see spectrum of plain GLYMO in Figure S6), cannot be observed. These results strongly suggest that the epoxy ring of GLYMO has exhaustively reacted with linseed oil or with the linseed oil/colophony mixture, while the methoxysilane functions are still unaltered (at least partially) within the experimental conditions used for functionalization.

**Figure 5:**
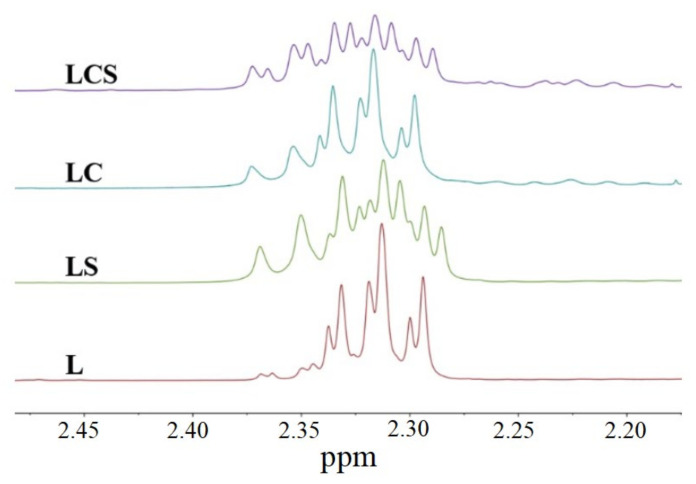
^1^H NMR spectra in CDCl_3_ of plain and functionalized materials. Enlargement of the signals between 2.25 and 2.40 ppm.

The different splitting of multiplets between 2.25 and 2.4 ppm in the spectra of the examined materials is also worth noting (Figure 5). As already mentioned, this signal is ascribed to methylene in the α position to ester carbonyl and is expected to be very sensitive to any change undergone by the ester group. The variations observed both when linseed oil is combined with colophony (LC) and with GLYMO (LS and LCS) suggest that the carboxyl group of fatty acids has been partially involved in the reaction with resin components and/or the epoxysilane cross-linker. 

**Figure 6:**
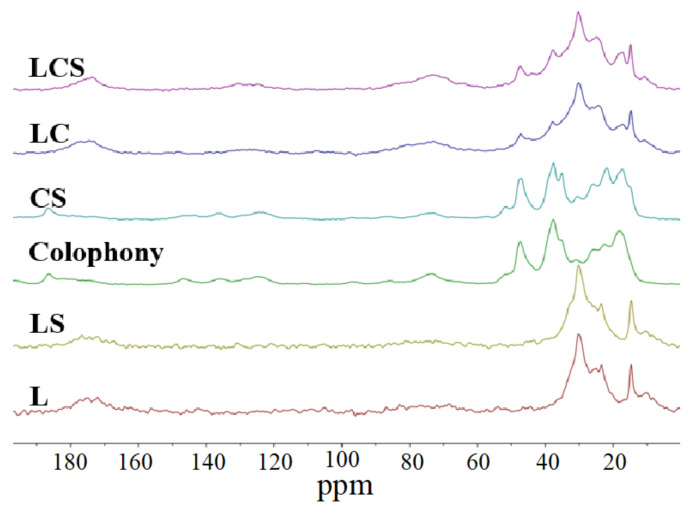
^13^C CPMAS NMR spectra of the original and cross-linked solid products.

^13^C CPMAS NMR spectra of the original and functionalized materials measured in the solid state are reported in Figure 6. In the case of L, LS, LC, and LCS, spectra were recorded after the curing process.

No significant differences in the chemical shift and in the shape of signals can be observed when comparing the spectra of both plain and functionalized linseed oil after curing (L and LS). This can be explained taking into account the low % of added GLYMO. The only observable difference between C and CS is the different relative intensity of the signals around 20 ppm, probably due to the higher proton density provided by the GLYMO. 

It is worth noting that in the spectra of samples LC and LCS the signal at 186 ppm, due to the carboxylic groups of the colophony, are not present. This is in agreement with the hypothesis of a “trans-esterification” process involving colophony and diglyceride species during the heating treatment [25,33].

The values of the spin-lattice relaxation times in the rotating frame T_1ρ_H and T_1ρ_C of varnishes LC and LCS are reported in Table 2. For the determination of both T_1ρ_H and T_1ρ_C spin-lattice relaxation in the rotating frame, the signals at 38 and 30 ppm were chosen in order to obtain the best results in the fitting process. These two signals are due to the C-1 and C-14 of the abietic acid (colophony component) and to the methylene groups of the linseed oil fatty acid chains, respectively.

The two components in both materials have equal values of T_1ρ_H. This indicates that the spin diffusion process makes the two material mixtures homogeneous in the tens of the Ǻ scale. On the contrary, the relaxation time values in the rotating reference system T_1ρ_C observed for varnish LC and LCS are considerably different. The spin-lattice relaxation times in the rotating reference system T_1ρ_C, unlike those of the proton (T_1ρ_H), are not influenced by the spin diffusion phenomenon, and therefore they reflect local mobility at the level of a few monomer units [42]. From the analysis of the T_1ρ_C values, an increase in the values for both components of the mixtures in the presence of the cross-linking agent (GLYMO) is clearly evident. This indicates that local rotational motions of the monomer units are less favored in the presence of the cross-linker. This phenomenon causes, at the macroscopic level, an increase in the glass transition temperature, as already observed in the DSC experiments.

### 3.3. Characterization of Coated Wood Specimens

The properties of LC and LCS were also comparatively investigated after their application to wood specimens and further curing. Particularly, their behavior was examined before and after artificial aging to evaluate the effect of GLYMO functionalization in terms of post-application performance. For this purpose, the following measurements were performed before and after the aging processes: chromatic variation and contact angle measurements, SEM-EDS, micro-FTIR, NMR, and hardness.

#### 3.3.1. Color Measurements

The chromatic variations of the samples were investigated to analyze any differences in color due to the modification of the Stradivari varnish before and after aging. The colorimetric measurements were carried out on the coated wood specimens. The parameters L*, a*, b*, and the global color variation ΔE* were obtained for the varnishes before and after aging cycles, and all the given results were average values from 15 different measurements, as explained in the Section 2.

**Figure 7:**
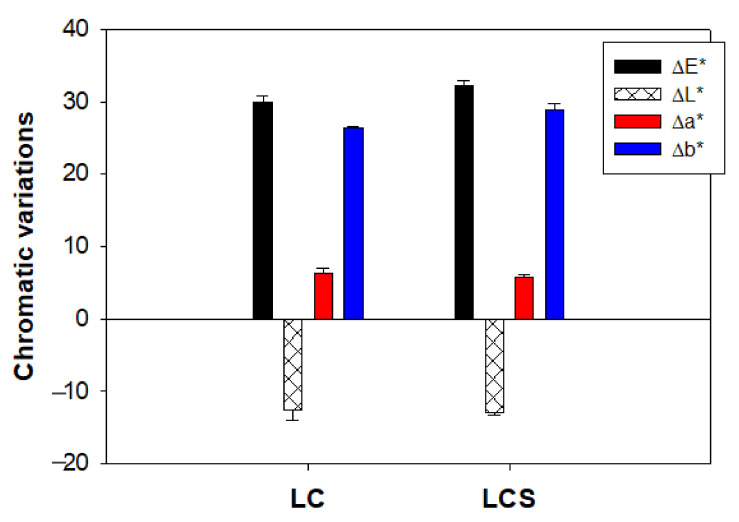
Chromatic variations induced by varnish when applied to wood specimens (changes compared to untreated wood).

After the application of varnishes, the wood surfaces undergo quite comparable overall chromatic changes (ΔE* referred to the original wood color = 30.0 ± 0.9 and 32.2 ± 0.8 for LC and LCS, respectively), suggesting that the modification induced by GLYMO and the consequent additional cross-linking do not significantly alter the original chromatic properties of the Stradivari varnish. GLYMO is a colorless clear liquid and the addition of a small amount as a cross-linking agent is not expected to significantly change the original color of Stradivari’s varnish. The color variation is mainly due to the strong increase in the b* coordinate (related to the blue-yellow changes), while coordinates L* (lightness) and a* (green-red changes) experience more limited variations (Figure 7). 

Aging processes induce mainly an increased yellowing of the coated specimens, which is more noticeable in the case of varnish LC and can be due to the degradation of the original components (e.g., oxidation of linseed oil and colophony). In fact, after irradiation by UV as well as after heat treatment, the coated wood surface undergoes further variations of chromatic coordinates, which are larger in the case of LC than the LCS varnish. Specifically, the values of Δb* (changes referred to the unaged coated surface, directly related to the yellowing) observed for LC (Δb*_UV_~18; Δb*_heat/hum_~22) are more than double (>100%) if compared to LCS (Δb*_UV_~9; Δb*_heat/hum_~7, Table 3). Consequently, the overall chromatic change, determined as the ΔE* value and taken as an indicator of the aging suffered by the varnishes, is much lower in the case of the modified varnish LCS, in agreement with the experimental results obtained from the analyses performed on varnish film samples. 

#### 3.3.2. Contact Angle Measurements

The results of the contact angle (α) measurements (average values from 15 measurements) showed that varnish LCS exhibits distinctly higher hydrophobic properties than varnish LC. In fact, the observed α increases by 34° (+34.7%) due to the structural modifications induced by GLYMO (Table 4). The more pronounced water-repellent behavior of modified varnish is preserved even after aging processes. In fact, the contact angle for the LC material drops at values lower than 90° after UV exposition or heat/humidity cycles, while α is still around 110° after both aging processes for LCS, indicating that the hydrophobic behavior of the surface is nearly preserved [43,44]. Hence, the coating obtained by varnish LCS provides a better performance, even in terms of protection from water.

#### 3.3.3. SEM-EDS Experiments

**Figure 8:**
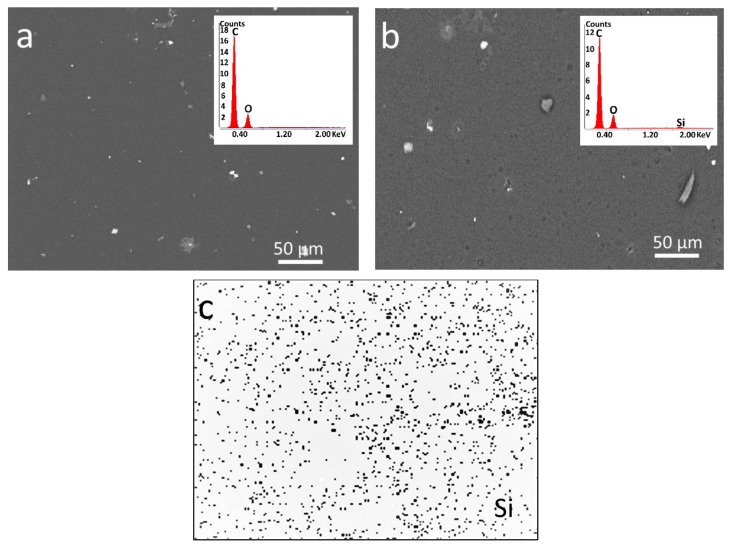
SEM-EDS images of coated wood specimens before aging processes: (**a**) varnish LC (EDS spectrum in the inset); (**b**) varnish LCS (EDS spectrum in the inset; (**c**) EDS mapping for element Si of varnish LCS (same area as (**b**)).

SEM-EDS analyses were performed to examine the surface morphology and chemical composition of aged and unaged samples. SEM images of the surface of varnishes LC and LCS before starting the aging processes are reported in Figure 8a and Figure 8b, respectively. The surfaces of both samples are quite homogeneous, with inclusions of small aggregates, probably due to dust deposits on the specimens. EDS spectra (insets in Figure 8a,b) are in agreement with the expected elemental compositions. In fact, only peaks of C and O are observed in the spectrum of varnish LC, while the peak of Si is also present in the case of LCS, due to the silane cross-linker being incorporated into the modified varnish. The presence of silicon, detected by the semi-quantitative analysis, is further confirmed by the EDS mapping experiment (Figure 8c), which shows the quite homogeneous dispersion of the Si element in the entire examined area of the film surface. The regular distribution of silicon is an indication that GLYMO has been homogeneously dispersed into the varnish matrix and that the cross-linking induced by epoxysilane has evenly affected the coating components.

**Figure 9:**
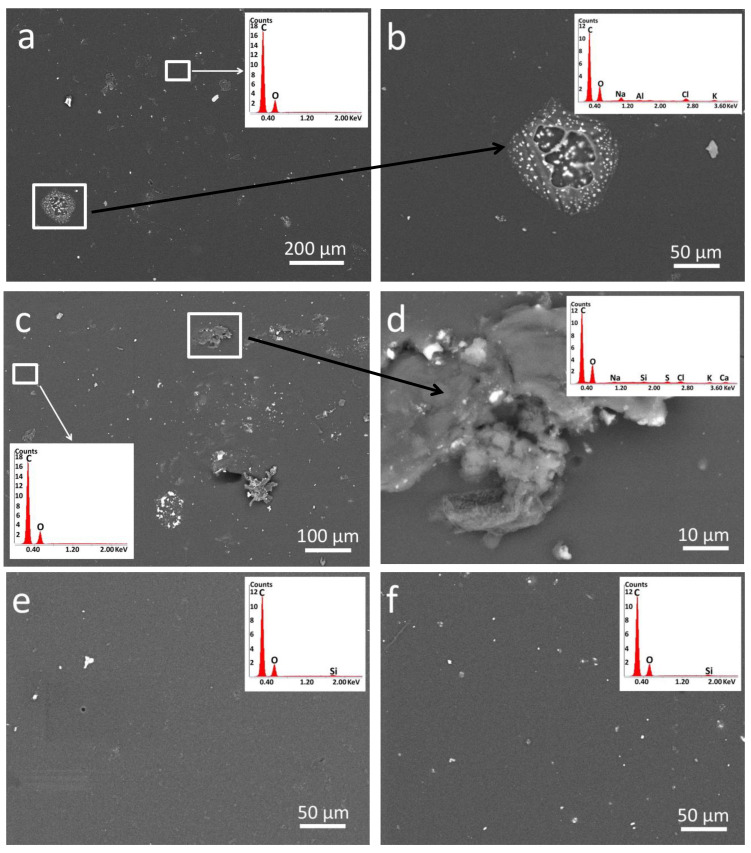
SEM images of coated wood specimens after aging processes: (**a**) varnish LC_UV (EDS spectrum in the inset); (**b**) magnification of damaged areas of LC_UV surface (EDS spectrum in the inset); (**c**) varnish LC_T (EDS spectrum in the inset); (**d**) magnification of damaged areas of LC_T surface (EDS spectrum in the inset); (**e**) varnish LCS_UV (EDS spectrum in the inset); (**f**) varnish LCS_T (EDS spectrum in the inset).

Figure 9a reports the SEM image of the LC coating after exposition under a UV lamp. The polymer layer is damaged and displays several inhomogeneous areas (Figure 9b), as also observed by the optical microscope. Varnish composition (C and O only) was confirmed in not-damaged areas, while in damaged areas EDS spectra showed the presence of different elements (e.g., Na, Mg, K, Ca) that could be ascribed to the wood substrate, whose surface is partially uncovered after artificial aging (inset in Figure 9b).

SEM observations showed the presence of several inhomogeneous areas on the specimens coated with LC, also after thermal aging (Figure 9c). In this case, too, the damaged areas display an elemental composition that includes some elements related to the uncovered wood surface (Figure 9d and corresponding inset). 

On the contrary, the surface of specimens coated with LCS appears quite homogeneous after both UV and thermal aging processes (Figure 9e,f). The elemental composition of varnish is preserved in all the observed areas and is similar to that before aging.

These results confirm that LC varnish undergoes considerable degradation during aging, which compromises its protecting properties. On the contrary, the silane-modified LCS varnish is more resistant to degradation induced by artificial aging, with the consequent preservation of its aesthetical as well as protecting features. 

#### 3.3.4. Micro-FTIR Measurements

**Figure 10:**
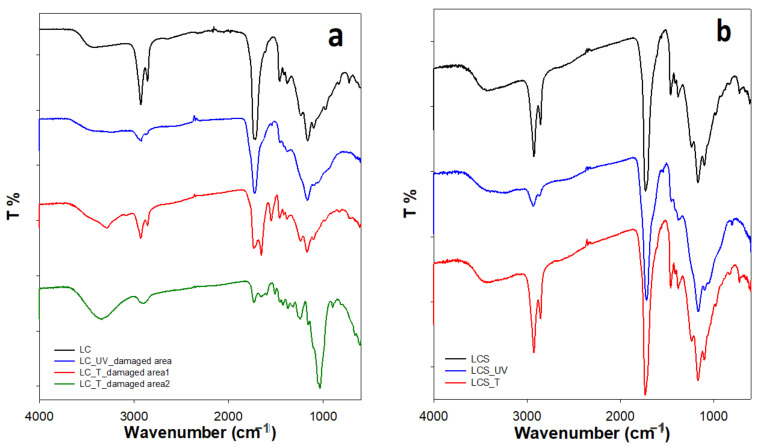
Micro-FTIR spectra of two different varnishes before and after aging processes: (**a**) varnish LC (different areas); (**b**) varnish LCS.

Wood samples coated with LC and LCS varnish were also investigated by µ-FTIR (attenuated total reflection, ATR mode) before as well as after aging processes. The two varnishes applied on wood surfaces display very similar spectra (Figure 10) when examined before any aging. On the contrary, the spectra of plain and modified varnish measured at the end of the artificial aging cycles display significant differences, in agreement with their already observed different behaviors towards artificial weathering. In particular, the spectra of LC recorded in correspondence with the damaged areas show several changes, particularly after the thermal aging cycle. For instance, several bands of the fingerprint region decreased their intensity or disappeared, while the carbonyl band centered at 1720 cm^−1^ became broader or was split into more bands due to the formation of different carbonyl derivatives (Figure 10a). In some cases, the spectra obtained on strongly damaged areas are very similar to the spectrum taken on the surface of untreated wood (for comparison, see Figure S7), confirming that weathering can strongly compromise the protecting properties of the plain LC coating and that some wood areas become uncovered (and unprotected) at the end of the thermal aging cycle. 

The spectra taken on different areas of the LCS surface after both artificial aging processes are very similar to the spectrum of the unaged material (Figure 10b), as the differences, if any, are very limited.

Results provided by the μ-FTIR analyses are in good agreement with the above-mentioned data obtained using optical microscope, SEM-EDS, and TGA-DSC experiments, confirming that the chemically modified varnish, LCS, displays an enhanced resistance to decay when compared with the traditional “Stradivari varnish” (LC). 

#### 3.3.5. Pencil Hardness Test

The hardness of wood coatings is an important parameter to evaluate the effectiveness of their protection from mechanical decay such as scratching. To evaluate the hardness properties of the investigated varnishes, pencil tests were performed on wood specimens coated with LC and LCS as well as on the corresponding coating films (prepared on glass slides) according to the standard method [21]. The considered scale for the test ranged between 9H (hardest) and 9B (softer), and the intermediate hardness level was “F”. This test provides information about the performance of coatings concerning resistance to mechanical decay and durability [45,46]. Table 5 reports the results of the pencil test performed on LC and LCS both before and after artificial aging. All the reported results are average values of three measurements performed on coated specimens and on coating films.

The results obtained from the tests carried out before the aging processes on the specimens coated with LC and LCS show that the modified varnish film is harder (H) than traditional varnish (B). This confirms again that cross-linking induced by epoxysilane provides a varnish layer that is more resistant to scratches than the LC varnish. Although a decrease in hardness level (softening) is observed after aging for both materials, the resistance of the not-modified varnish is affected to a larger extent than the functionalized one. In fact, the hardness of LC decreases by 2–4 levels depending on the aging process (from B to 2B-3B and to 3B-4B after UV and thermal aging, respectively), while a reduction by only 1–2 levels is observed for the hardness of LCS (from H to F and B after UV and thermal aging, respectively). Interestingly, the modified varnish after aging displays a hardness level (F or HB) that is still higher than the not-aged traditional varnish (B). Hence, the results of pencil test confirm again that additional cross-linking induced by GLYMO in the linseed oil/colophony mixture provides an improved resistance of the resulting varnish to scratches, even after artificial aging, if compared to the traditional “Stradivari” varnish.

## 4. Conclusions

This research work focused on the improvement of the properties of Stradivari’s varnish using proper chemical modification. Limited amounts of the cross-linking agent 3-glycidyloxypropyltrimethoxysilane (GLYMO) were added to the traditional linseed oil/colophony mixture (75:25), which is still used to prepare the varnish for contemporary bowed musical instruments. Epoxysilane reacts through the epoxide ring with both components of the varnish, introducing reactive alcoxysilane groups into the oil/resin matrix. In addition to the known curing of the varnish induced by the oxidation of fatty acid chains and consequent radical reactions, a cross-linking due to the condensation of the silane group, therefore, can take place. 

As a consequence, the modified Stradivari’s varnish is more resistant to mechanical decay (e.g., scratches), as shown by pencil hardness tests (the hardness changes from B to H). Moreover, increased resistance to decline induced by aging was demonstrated using comparative investigations carried out on the plain and modified varnishes before and after artificial laboratory aging (cycles of UV irradiation and temperature/humidity variations). Results obtained using different techniques (OM, SEM-EDS, TGA and DSC, FTIR, and μ-FTIR) coherently showed that the GLYMO-modified material is definitely less affected by aging than the traditional Stradivari’s varnish. For instance, the traditional varnish, LC, undergoes a drastic T_g_ decrease after artificial aging (from 17.4 to −26.3 or −28.2, depending on the aging process), while the T_g_ reduction suffered by the modified material, LCS, was distinctly smaller (from 40.3 to 33.5 or 25.5, depending on the aging process). This suggests that the modifications induced by GLYMO make the varnish less soft than the plain material and better able to preserve its properties after artificial aging.

Interestingly, the chemical modification does not induce significant changes in the chromatic properties of the varnish (ΔE* was about 30 and 32 for LC and LCS, respectively, referring to the original wood color) Moreover, the color changes, particularly the yellowing, induced by artificial aging are definitely smaller in LCS than in LC. The modified varnish displays increased water-repellence when compared to the traditional one (by about 34%), and the hydrophobic behavior is also preserved (contact angle value around 110°) after performing artificial aging. On the contrary, wood samples coated with traditional Stradivari’s varnish display a quite hydrophilic surface (α < 90°) after aging.

In conclusion, the modified Stradivari’s varnish represents a promising alternative to the one prepared according to the traditional method of contemporary violin-makers. In fact, it retains the unique aesthetical features of the historical finish, and, at the same time, is more resistant to mechanical and physico-chemical decay. 

## Figures and Tables

**Table 1:** Results of DSC measurements.

Films	T_g_ (°C)
Pure linseed oil (L)	−6.8
Functionalized linseed oil (LS)	31.5
Colophony (C)	60
Functionalized colophony (CS)	75
Varnish LC	17.4
Varnish LC after UV aging (LC_UV)	−28.3
Varnish LC after thermal aging (LC_T)	−26.2
Varnish LCS	40.3
Varnish LCS after UV aging (LCS_UV)	33.5
Varnish LCS after thermal aging (LCS_T)	25.5

**Table 2:** Values of the spin-lattice relaxation times in the rotating reference systems T_1ρ_H and T_1ρ_C of the varnishes.

Samples	LC	LCS	LC	LCS
Ppm	T_1ρ_H (ms)		T_1ρ_C (ms)	
38 (colophony)	0.9 ± 0.1	1.3 ± 0.1	6.4 ± 0.1	9.6 ± 0.1
30 (oil)	0.8 ± 0.1	1.2 ± 0.1	2.3 ± 0.1	3.8 ± 0.1

**Table 3:** Chromatic variations of treated wood samples after two different aging cycles (changes compared to unaged specimens).

Samples	UV-Aged (720 h)				Heat/Humidity-Aged (720 h)			
	ΔE*	ΔL*	Δa*	Δb*	ΔE*	ΔL*	Δa*	Δb*
Varnish LC	21.9 (±5.8)	−9.1 (±5.5)	8.1 (±2.6)	18.0 (±3.1)	23.5 (±0.4)	−5.9 (±1.2)	6.0 (±0.9)	21.9 (±0.2)
Varnish LCS	11.9 (±2.3)	−5.5 (±2.9)	4.8 (±0.8)	9.2 (±1.3)	15.2 (±0.1)	−9.7 (±0.2)	9.3 (±0.3)	6.9 (±0.5)

**Table 4:** Contact angle measurements α (°) on coated wood specimens before and after aging processes.

Samples	Before Aging	UV-Aged	Heat/Humidity-Aged
		(720 h)	(720 h)
Varnish LC	98.5 (±8.0)	86.6 (±6.0)	82.0 (±2.7)
Varnish LCS	132.5 (±3.8)	110.8 (±1.6)	110.3 (±7.2)

**Table 5:** Results of pencil hardness test.

Samples	After Treatments	UV-Aged	Thermal-Aged
Varnish LC	B	3B-2B	4B-3B
Varnish LCS	H	F	HB

## Data Availability

The data presented in this research study are available in the present article and in the related Supplementary Information.

## Supplementary Materials

The following supporting information can be downloaded at: https://www.mdpi.com/article/10.3390/polym15173652/s1, Figure S1. FTIR spectrum of 3-glycidyloxypropyltrimethoxysilane (GPTMS or GLYMO); Figure S2. Thermogram (TGA) of Linseed oil (L) and functionalised Linseed oil (LS); Figure S3. Thermogram (TGA) of colophony (C) and functionalised colophony (CS); Figure S4. ^1^H NMR spectra in CDCl_3_ of plain and functionalised materials: enlargements of specific spectral regions; Figure S5. ^1^H NMR spectra in CDCl_3_ of LC and LCS (selected regions). Colophony spectrum is also reported for comparison; Figure S6. ^1^H NMR spectrum in CDCl_3_ of 3-glycidyloxypropyltrimethoxysilane (GPTMS or GLYMO); Figure S7. Micro-FTIR Spectrum (ATR mode) of plain untreated wood.
