# Supplementary material for: Stradivari’s Varnish Revisited: Feature Improvements Using Chemical Modification
Source: Polymers (Basel). 2023 Sep 4;15(17):3652. doi: 10.3390/polym15173652 (PMC10490378; doi:10.3390/polym15173652)
Supplement: Supplementary file 1 [file polymers-15-03652-s001.zip › polymers-2573335-supplementary.pdf]

Stradivari varnish revisited: feature improvements by chemical modification

Maduka L. Weththimuni,[1,2*] Giacomo Fiocco,[2,3] Chiara Milanese,[1] Alberto Spinella,[4] Maria Luisa Saladino,[5] Marco Malagodi[2,3], Maurizio Licchelli[1,2*]

[1]Department of Chemistry, University of Pavia, via T. Taramelli 12, 27100 Pavia, Italy

[2]CISRiC, University of Pavia, via A. Ferrata 3, 27100 Pavia, Italy

[3]Department of Musicology and Cultural Heritage, University of Pavia, Corso Garibaldi 178, 26100, Cremona, Italy

[4]Advanced Technologies Network Center, University of Palermo, Viale delle Scienze, Ed. 18, 90128, Palermo, Italy

[5]Department of Biological, Chemical, and Pharmaceutical Sciences and Technologies (STEBICEF), University of Palermo, Viale delle Scienze, Ed. 17, 90128, Palermo, Italy

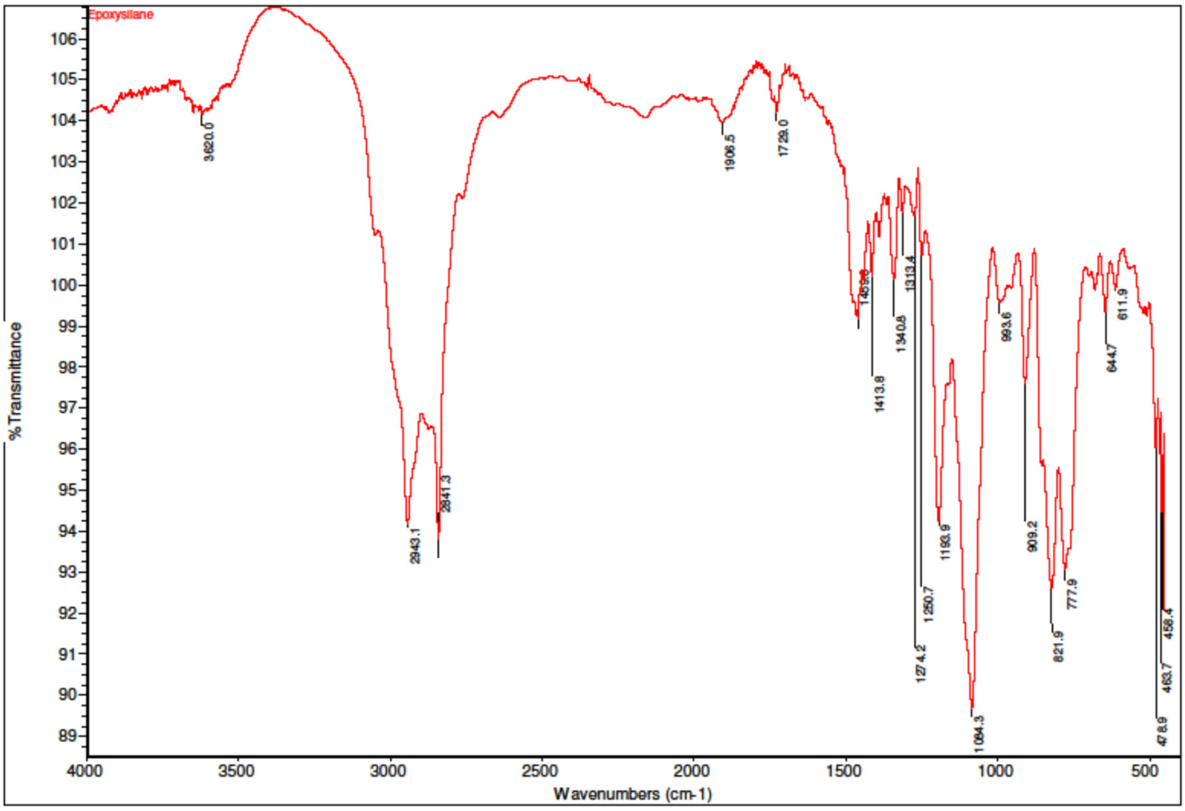

Figure S1. FTIR spectrum of 3-glycidyloxypropyltrimethoxysilane (GPTMS or GLYMO)

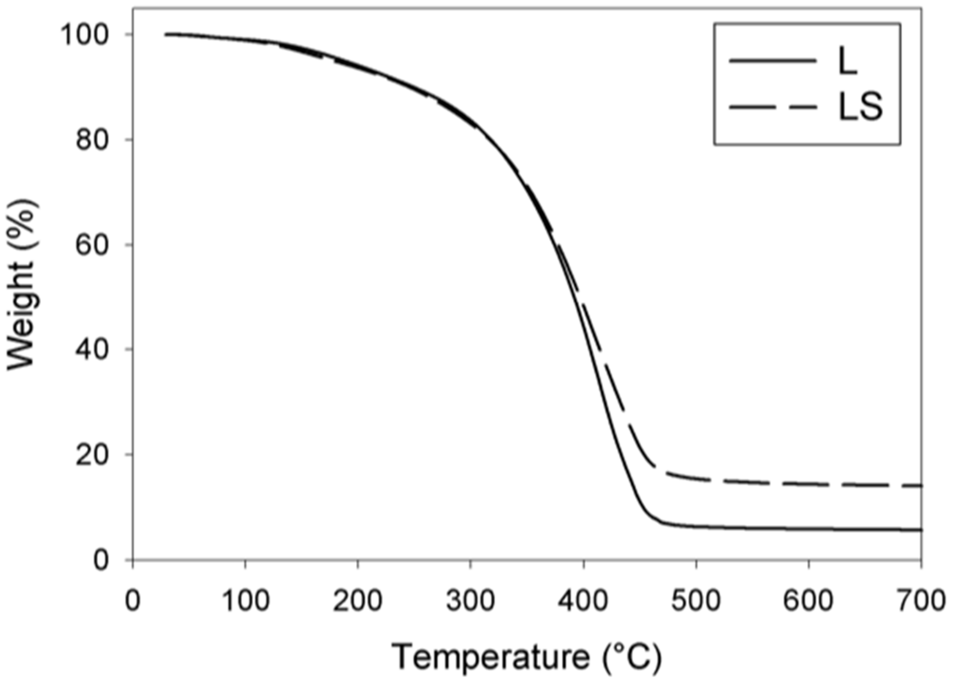

Figure S2. Thermogram (TGA) of Linseed oil (L) and functionalised Linseed oil (LS)

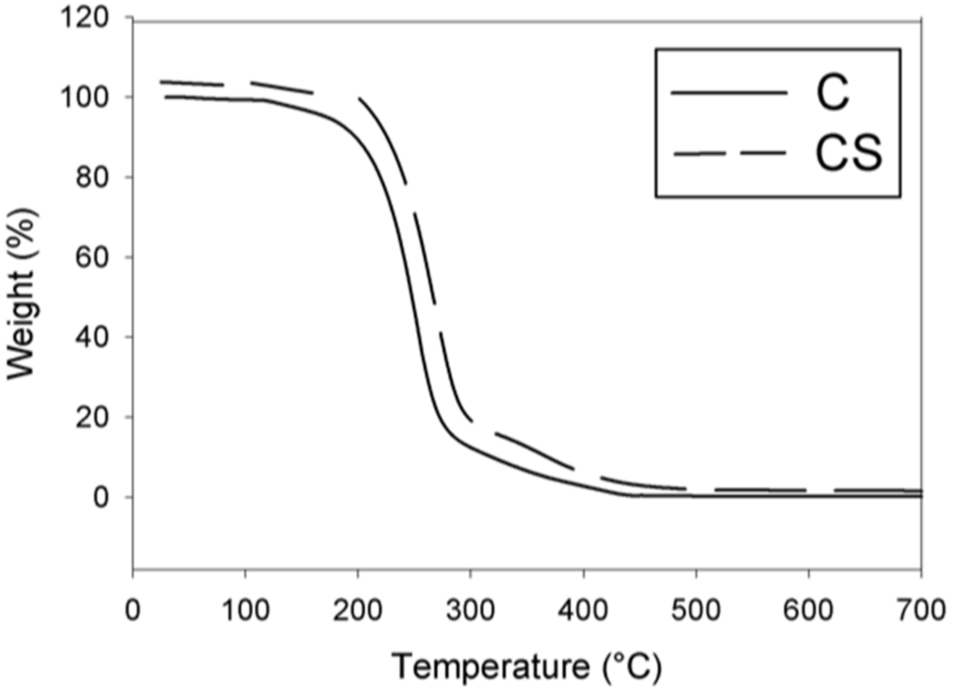

Figure S3. Thermogram (TGA) of colophony (C) and functionalised colophony (CS)

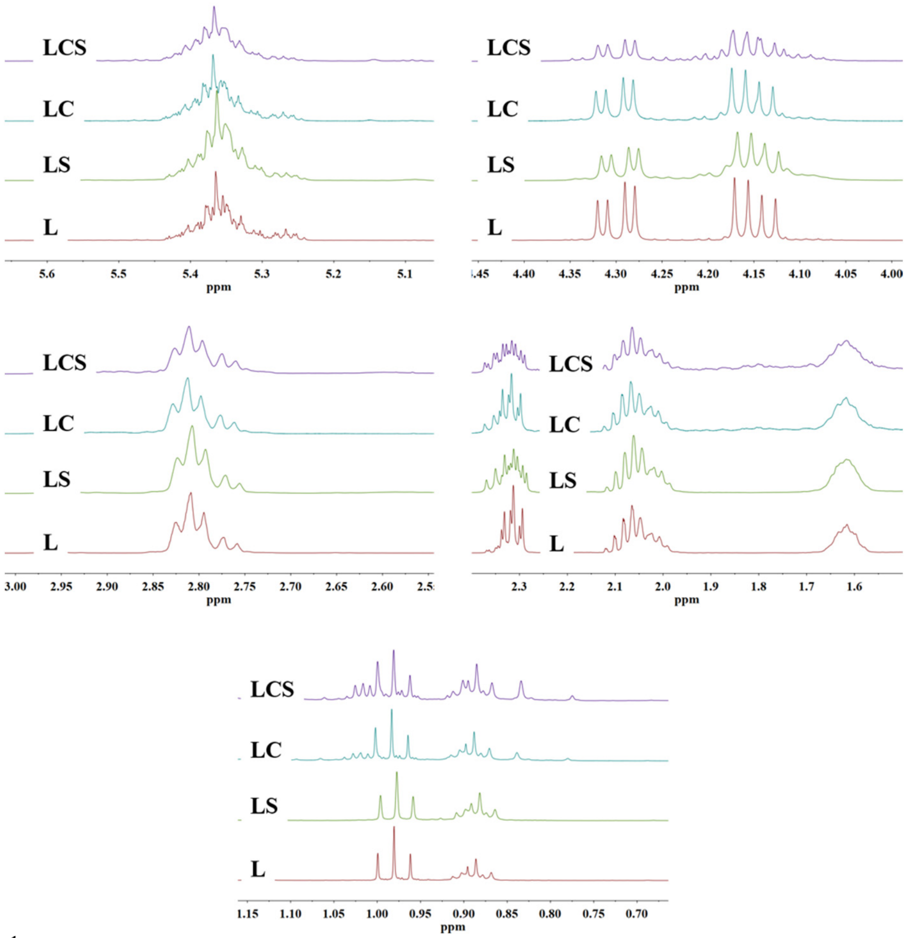

Figure S4. ^{1}H NMR spectra in $CDCl_3$ of plain and functionalised materials: enlargements of specific spectral regions

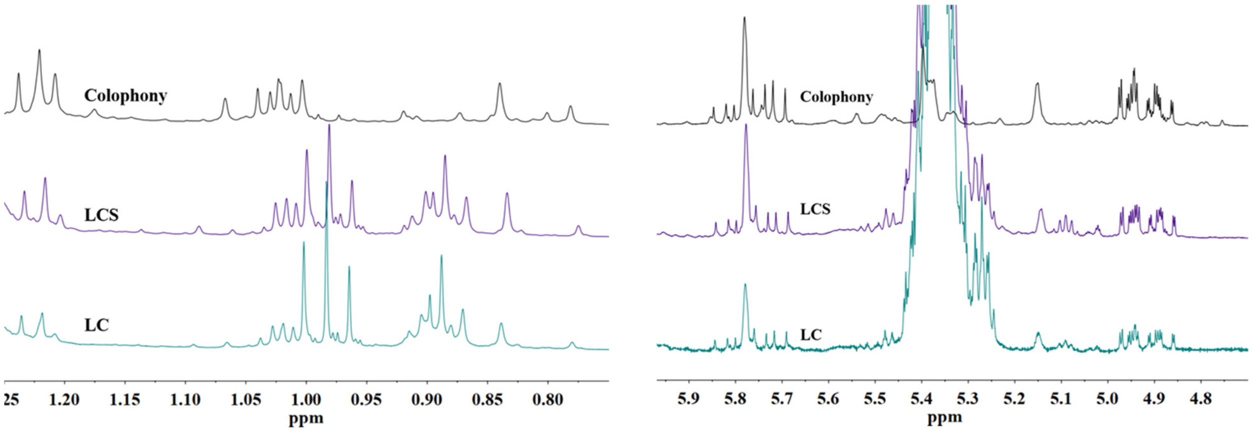

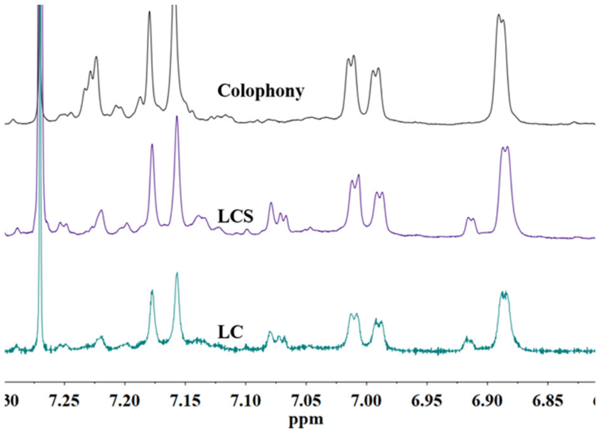

Figure S5. ^{1}H NMR spectra in $CDCl_3$ of LC and LCS (selected regions). Colophony spectrum is also reported for comparison

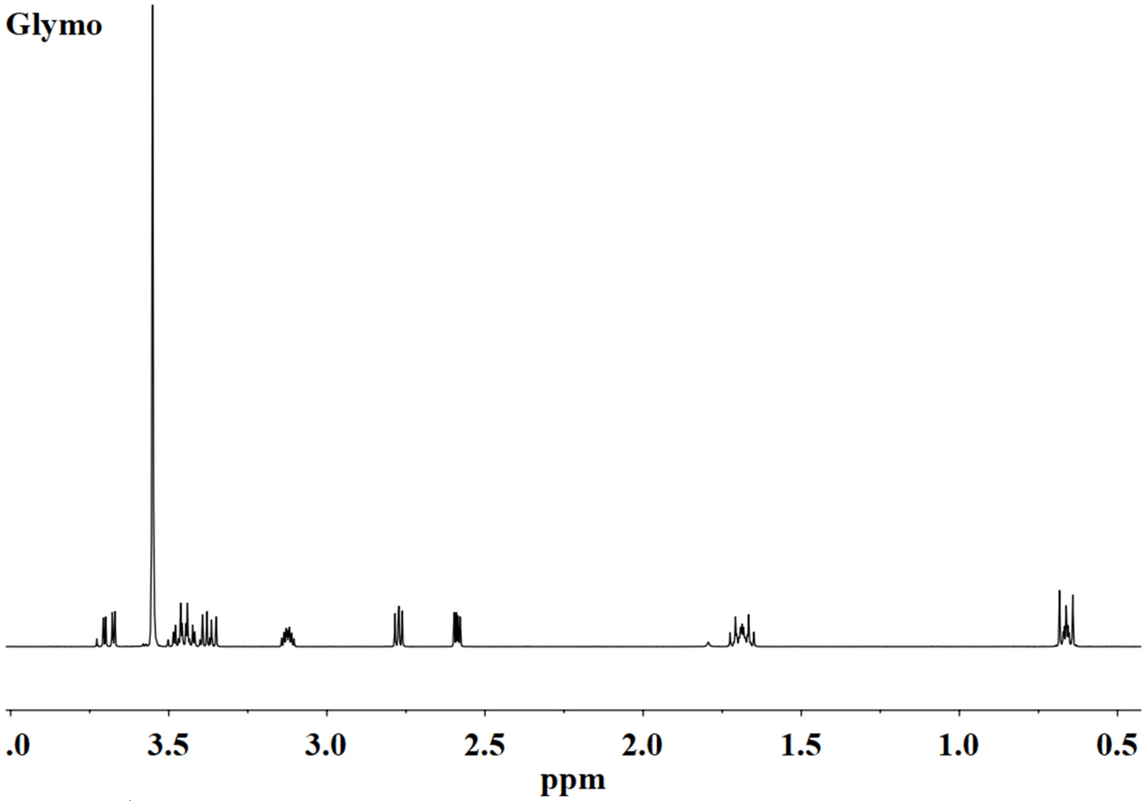

Figure S6. ^{1}H NMR spectrum in $CDCl_3$ of 3-glycidyloxypropyltrimethoxysilane (GPTMS or GLYMO)

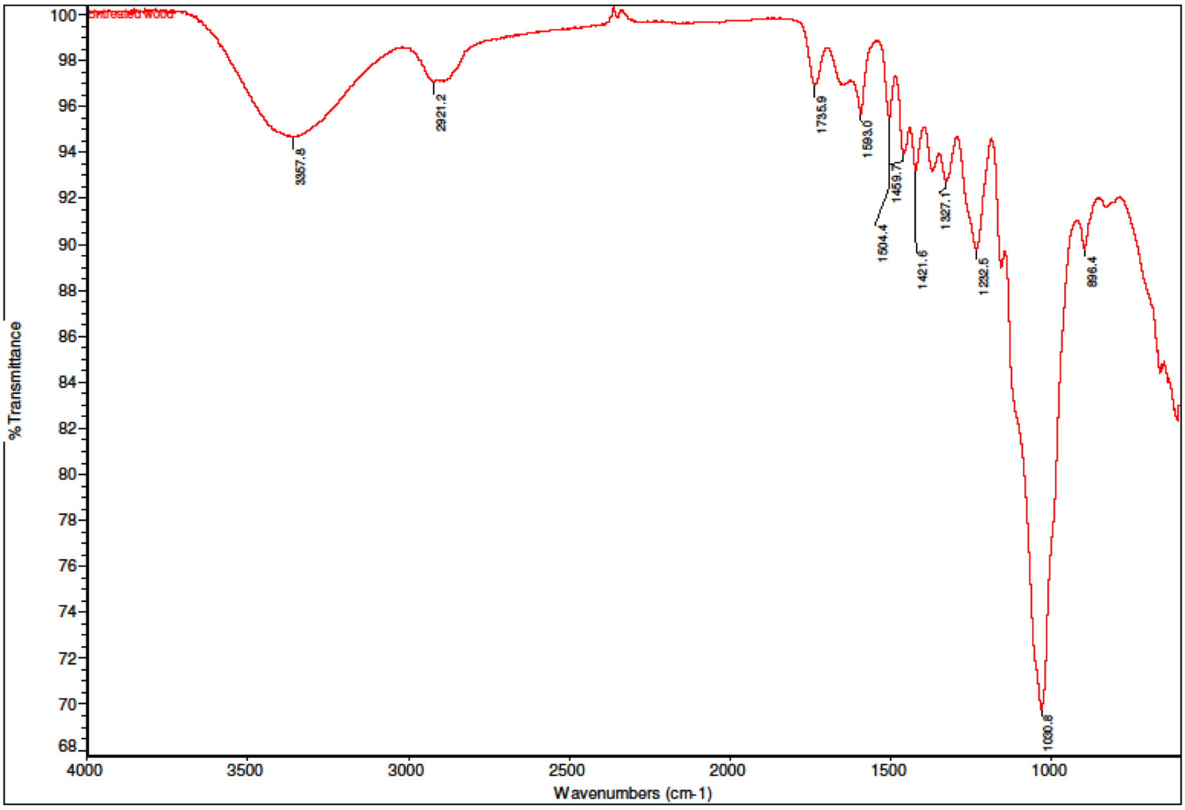

Figure S7. Micro-FTIR Spectrum (ATR mode) of plain untreated wood

Accuracy data and/or performance features of the instruments:

Konica Minolta (Konica Minolta, Inc., Tokyo, Japan) spectrophotometer and the Model is CM-2600d; Spectral Reflectance: Standard deviation within 0.1% (360 to 380 nm within 0.2%) Colorimetric Value: Standard deviation within ΔE*ab 0.04 (Measurement conditions: White calibration plate measured 30 times at 10-second intervals after white calibration was performed.

PerkinElmer Spectrum 100 FT-IR instrument (Perkin-Elmer, Waltham, MA, USA), equipped with an universal ATR accessory (Diamond crystal): absolute transmission accuracy, specialized components to block stray reflections, advanced electronics for high fidelity signal sampling, high degree of control over source image allows optimization of measurement parameters, abscissa precision better than 0.01 cm^{-1}.

Nicolet iN10 Thermo Fischer μ-FT-IR spectrometer (Thermo-Fisher, Waltham, MA, USA) in ATR mode (Germanium crystal): Dynamically aligned high-speed interferometer; high-speed collection up to 10 scans per second at 16 cm^{-1} and 0.4 cm^{-1} maximum resolution (with Nicolet iZ10 FTIR external module). Most samples require just few seconds of collection time. Provides superior sensitivity for challenging samples and smallest particles. Signal-to-noise at 100 μm, 2100–2000 cm^{-1}, 4 cm^{-1}Resolution, 2 minutes, Better than 25,000:1 with cooled detector.
